# Development and validation of a Quality of Life and Treatment Satisfaction measure in feline osteoarthritis

**DOI:** 10.3389/fvets.2026.1774034

**Published:** 2026-08-07

**Authors:** Edwina Gildea, Jill Thompson, Alasdair Cook, George Skingley, Amy Findley, Charlotte Panter, Thaisa Lucas Sandri, Aoife Mahon-Smith, Aoife Lydon

**Affiliations:** 1 Global Medical Affairs, Zoetis Inc., Parsippany, NJ, United States; 2 Digital, Data and Analytics, Zoetis Inc., Parsippany, NJ, United States; 3 Veterinary Health Innovation Engine (vHive), University of Surrey, Guildford, United Kingdom; 4 Patient-Centered Outcomes, Adelphi Values Ltd., Bollington, United Kingdom

**Keywords:** cat, feline, osteoarthritis, owner, pain, pain management, Quality of Life, Treatment Satisfaction

## Abstract

**Introduction:**

Osteoarthritis (OA) in cats causes pain and mobility impairment, negatively impacting quality of life (QoL) of cats and their owners. Owner-perceived treatment satisfaction can influence treatment adherence and subsequent health outcomes for the cat. Currently, no OA-specific instrument for cats assesses all three concepts. This study aimed to address this gap by developing and psychometrically evaluating an owner-completed feline OA-specific measure—the “Feline OA Quality of Life and Treatment Satisfaction Assessment” (FeOA-QoL-TS-A)—to measure cat QoL, owner QoL, and owner treatment satisfaction.

**Methods:**

A mixed-methods approach was used. The draft FeOA-QoL-TS-A was developed using a conceptual model derived from an earlier meta-synthesis of published literature and supplementary review. Qualitative interviews were then conducted with eight owners of cats with OA to evaluate the content validity of the draft FeOA-QoL-TS-A. The revised FeOA-QoL-TS-A was then administered to owners of OA-treated cats (*n* = 139) at six timepoints during a phase 4 field study to evaluate the psychometric properties of the resulting item-scale structure.

**Results:**

Interview findings informed revisions to the draft 25-item FeOA-QoL-TS-A, which was well understood by owners. Item-level analysis and confirmatory factor analysis supported the removal of five items and confirmed the structure of three distinct domains: Cat QoL, Owner QoL, and Treatment Satisfaction. The final 20-item FeOA-QoL-TS-A demonstrated good internal consistency and test–retest reliability. Convergent validity was supported by strong correlations with concurrent measures. Known groups comparisons provided strong evidence that the instrument can differentiate between groups with varying QoL levels. Ability to detect improvement in Cat and Owner QoL domains was demonstrated through statistically significant improvements over time, with larger effect sizes reported for the ‘improved’ group compared with the ‘stable’ group. Anchor-based analyses supported −1.0 and −0.9-point responder definitions for Cat and Owner QoL domains, respectively.

**Conclusion:**

The final 20-item FeOA-QoL-TS-A is a valid, reliable, and responsive instrument for assessing OA-specific cat QoL, owner QoL, and treatment satisfaction. Its flexible domain structure supports application in veterinary practice, stakeholder communications, and clinical research endpoints. The FeOA-QoL-TS-A addresses a critical measurement gap by providing a comprehensive and practical solution to evaluate the multifaceted impacts of feline OA.

## 1 Introduction

Osteoarthritis (OA) in cats is a chronic progressive condition characterized by the deterioration of cartilage, causing pain (acute and chronic) and mobility impairment (1). These symptoms negatively impact the quality of life (QoL) of affected cats, including their ability to perform normal daily activities (e.g., jumping, climbing stairs, playing, exploring, grooming, and accessing the litter box), which may lead to reduced independence and engagement with their environment (2, 3). Over time, these functional limitations may contribute to secondary changes such as altered body weight or body condition, reduced muscle condition through disuse, and progressive functional decline, thereby reinforcing a cycle of pain, inactivity, and worsening physical function (4, 5). This supports that OA should be considered both a musculoskeletal disease and a pain condition, with broad effects on cats’ physical comfort, behavior, emotional wellbeing, social interaction, and wider QoL (2, 3).

OA is common in cats, with its prevalence increasing with age; studies have shown that over 90% of cats aged 12 years or older had radiographic evidence of OA (6, 7). Due to their small size, natural agility, and instinct to protect themselves against predators, cats often mask signs of pain and discomfort (8, 9). OA-related discomfort may present as subtle behavioral changes, including reduced activity, increased resting, reluctance to move, irritability, withdrawal from social interaction, and changes in grooming or elimination habits (3, 10). Consequently, the clinical signs of OA are frequently under-recognized by owners and underdiagnosed by veterinarians. Early screening is essential to improve OA identification, enabling the establishment of a baseline for each cat and helping owners detect changes in their cat’s behavior or ability over time (11).

While radiography remains a key diagnostic tool, it is widely acknowledged that this does not always correlate with clinical signs, such as the degree of pain experienced (12–14). Therefore, veterinarians typically rely upon their clinical expertise, combining a thorough medical history obtained from the owner, observations of behavioral changes, and physical examination findings to reach a diagnosis and inform treatment decisions (10, 15). Existing OA-specific clinical metrology instruments and checklists are designed to measure the severity of functional impairment caused by OA in cats (12, 16, 17); however, they do not assess impaired cat QoL due to OA-related pain. A sensitive tool capable of detecting subtle changes in QoL could aid in diagnosis and, through early detection and intervention, mitigate the detrimental impact of OA on cat QoL (11). In the context of companion animals, QoL typically refers to the animal’s overall wellbeing as assessed by both objective indicators (i.e., observable clinical signs and functional impairment) and subjective components (i.e., owner-reported perceptions of the animal’s comfort, behavior, and emotional state) (18, 19).

Generic QoL instruments—such as the VetMetrica™ Feline Health-Related Quality of Life (HRQoL) instrument (20, 21), which is a validated HRQoL tool that has been used in cats with OA (22, 23)—are useful for broad assessments of QoL. However, as generic HRQoL instruments are designed to assess overall health status across conditions, they may be less sensitive to detect nuanced changes in QoL that are directly attributable to OA progression or treatment response (2, 22, 24). Furthermore, these instruments are not tailored to the specific challenges and symptomatology of cat OA and may not capture the full spectrum of OA-related impacts, including the owner’s QoL and experience/satisfaction with treatment, which are critical for adherence and long-term management.

In addition to impaired cat QoL, chronic diseases in companion animals, such as OA in cats, have been shown to negatively affect owner QoL (25). In this context, owner QoL refers to the overall wellbeing of the owner in relation to their pet’s health. This is typically assessed by evaluating owner-rated perceptions of the impact of caregiving, encompassing both the practical demands and subjective experiences, such as impacts to emotional (i.e., anger, guilt) and social (i.e., relationships with others) functioning (26). Owners of pets with chronic conditions report greater caregiver burden, which is associated with increased stress and impaired social functioning (25). Studies involving owners of dogs with OA have highlighted concerns such as anxiety around veterinary visits and challenges in managing pain (27, 28). Despite these findings, there is a notable gap in the literature regarding the impact of cat OA on owner QoL, and no existing validated OA-specific instrument in cats includes an assessment of this concept.

Management of OA-related pain in cats involves a multimodal approach (medication and non-medication therapies) to effectively address multiple components of OA and the needs of the individual cat and their owner (29, 30), which may be burdensome for owners. Owners’ adherence to a treatment regimen is influenced by owner treatment satisfaction (i.e., satisfaction with the treatment process and results), with lower adherence associated with lower treatment satisfaction (31, 32). Adherence is also influenced by owner burden, including treatment plan complexity (31), dosing regimen, consultation time, physical or social risks, and method of administration (33). Inadequate adherence can lead to deterioration in health outcomes (31), underscoring the importance of evaluating owner treatment satisfaction in OA management.

Given the established impact of OA in cats and the limited tools available to measure the impact of the condition, there is a need for an instrument in cat OA that assesses cat QoL, owner QoL, and owner treatment satisfaction. A standardized, OA-specific instrument would enable more precise monitoring of disease progression, facilitate individualized treatment planning, and provide robust endpoints for evaluating therapeutic efficacy in clinical trials. Such a tool would also support improved communication between veterinarians and cat owners, enhancing shared decision-making and ultimately improving outcomes for cats living with OA (34–36).

This study builds on prior research conducted by Zoetis and Manchester Metropolitan University, which developed a conceptual model of impaired QoL in cats with OA based on a meta-synthesis of published literature (2). Seven domains were identified: mobility (e.g., climbing), physical appearance (e.g., grooming), energy and vitality (e.g., activity levels), temperament (e.g., aggressiveness), pain expression (e.g., tolerating touch), sociability (e.g., interactions with others), and physical and mental wellbeing (e.g., toileting). A supplementary literature review and retrospective analysis of qualitative interviews with cat owners [from a previous study (37)] further explored the impact of cat OA on owner QoL and treatment satisfaction. Key themes included increased caregiving demands, emotional distress, reduced social interaction, and the influence of treatment factors such as efficacy, administration method, frequency, and cost (21, 25, 26, 38–40). These findings informed the development of the 29-item Feline Osteoarthritis Quality of Life and Treatment Satisfaction Assessment (FeOA-QoL-TS-A), designed to assess cat QoL, owner QoL, and treatment satisfaction. However, further instrument development was required, as described in this manuscript.

The aim of this study was to continue the development of the FeOA-QoL-TS-A, specifically to evaluate its content validity through qualitative interviews (Stage 1) and then evaluate its psychometric properties and estimate meaningful change thresholds using data from a phase 4 field study (Stage 2).

## 2 Materials and methods

### 2.1 Study design

**Figure 1:**
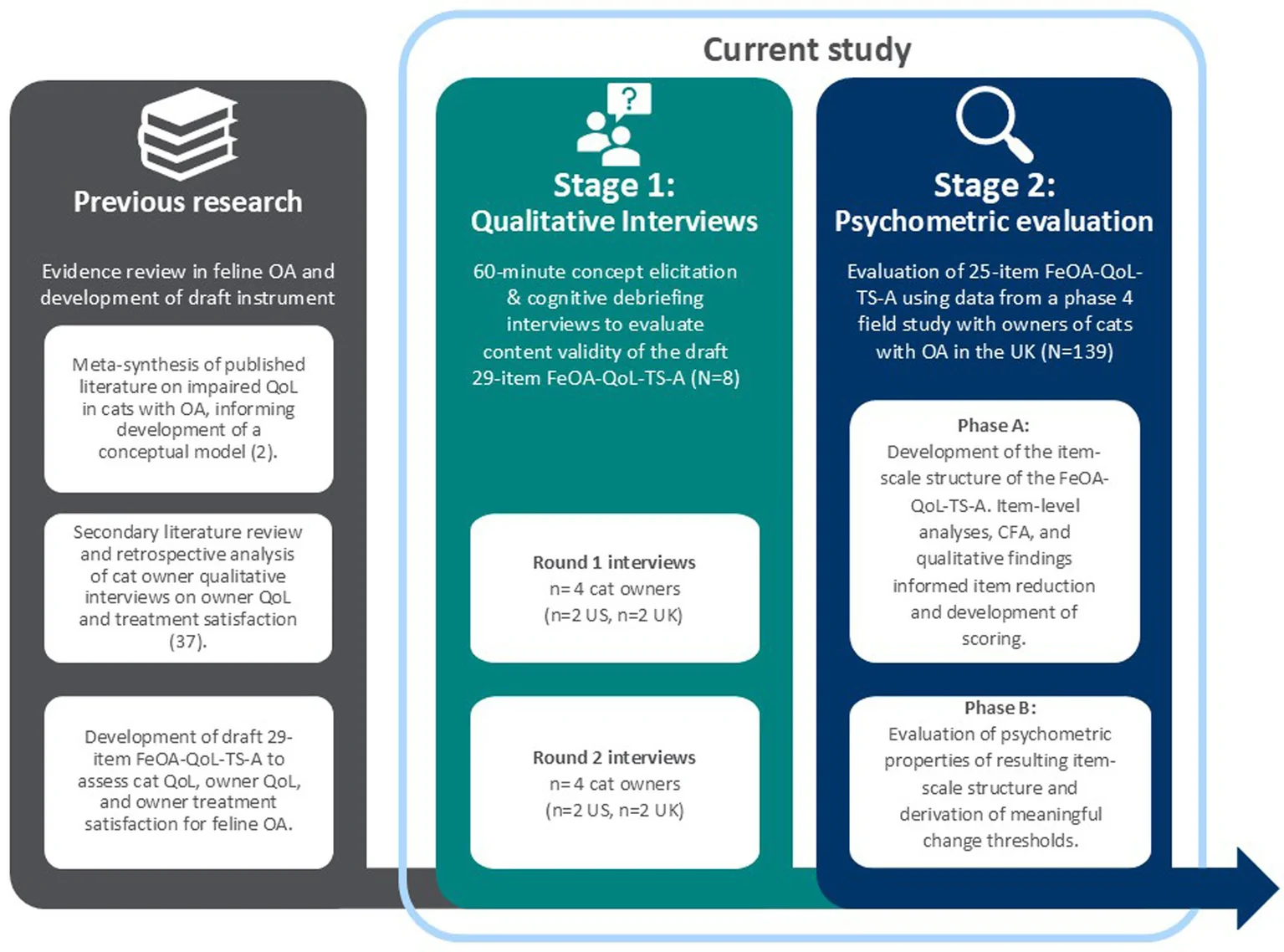
Overview of study activities.FeOA-QoL-TS, Feline steoarthritis Quality of Life and treatment satisfaction; OA, Osteoarthritis; QoL, Quality of life; US, United States; UK, United Kingdom. References: (2, 37).

This was a mixed-methods study, involving qualitative (Stage 1) and quantitative (Stage 2) data collection and analysis. Study activities were completed in two stages, as outlined in Figure 1.

- Stage 1 was a cross-sectional, non-interventional qualitative study, comprising 60-min combined concept elicitation (CE) and cognitive debriefing (CD) telephone interviews with owners of cats with a veterinarian-diagnosis of OA (*N* = 8), in the United States (US; *n* = 4) and United Kingdom (UK; *n* = 4). The interviews intended to explore owners’ experiences of caring for a cat with OA, inform any revisions to the previously developed conceptual model, evaluate the conceptual comprehensiveness of the draft 29-item FeOA-QoL-TS-A, and update, add or remove items ahead of psychometric evaluation. Interviews were conducted in two rounds to allow for modifications and testing of the updated instrument between rounds.
- Stage 2 comprised psychometric evaluation of the subsequently updated draft 25-item FeOA-QoL-TS-A. It used data collected in a multi-center, uncontrolled, prospective, longitudinal, non-interventional phase 4 field study of Frunevetmab (Solensia®; a treatment to control OA pain in cats) conducted in the UK (*N* = 139). This intended to inform item finalization, development of scoring, and evaluation of measurement properties, including the instrument structure and item-level analyses, reliability, convergent validity, known groups comparisons, ability to detect change over time, and assessment of meaningful change thresholds. Note, the results of the field study exploring the efficacy of Frunevetmab on QoL are presented in a separate manuscript (23).

All research was conducted in accordance with the World Medical Association’s Declaration of Helsinki and General Data Protection Regulation. Participants for Stage 1 provided written informed consent via Adobe Sign prior to commencing any study-related activities. Participants for Stage 2 indicated their consent by accepting the terms of the electronic clinical outcome assessment (eCOA). All participants were compensated for their time following completion of the study activities. Ethical approval was not required for the studies involving owners of animals in accordance with the local legislation and institutional requirements because of the methods and nature of the research conducted.

### 2.2 Clinical outcome assessments (COAs) included in the study

#### 2.2.1 The FeOA-QoL-TS-A

The owner-completed FeOA-QoL-TS-A was designed to assess three hypothesized domains: Cat QoL, Owner QoL, and Treatment Satisfaction. The instrument was refined based on round 1 and round 2 interview findings and was finalized following completion of all stages of the current study. Cat QoL and Owner QoL domain items instructed owners to indicate the degree to which each concept reflected the experience of their cat’s QoL/their QoL, respectively, over the ‘past 7 days’, using a 5-point response scale (‘Not at all’ to ‘A great deal’). Two Cat QoL domain items used a 6-point response scale with an additional ‘Not applicable’ option for owners who had not observed their cat perform an activity/behavior within the recall period. Two Cat QoL domain items were reverse scored to ensure conceptual alignment. Reverse scoring involves attributing numeric values in the opposite direction on the response scale to ensure conceptual alignment between items and consistent interpretation of the item scores that form the domain score. Treatment Satisfaction domain items instructed owners to think about their cat’s ‘most recently prescribed treatment for their arthritis’, using a 5-point response scale (‘Not at all’ to ‘Very much’). Five-point response scales were used to ensure adjacent response options were distinct and reflective of true differences in observable signs of OA in cats. A recall period of the ‘past 7 days’ was used to ensure owners had sufficient opportunity to observe OA symptom presentation (including changes in response to treatment), while being short enough to minimize recall error and respondent burden (41). The FeOA-QoL-TS-A included in the CD interviews was in pen and paper format; the instrument was migrated to an eCOA format for the phase 4 field study.

The FeOA-QoL-TS-A may not be used or altered in any way without prior written permission from Zoetis, and paper and smartphone formats are available for use under a formal licensing agreement with Zoetis. To request permission for use or for additional information and inquiries please contact the corresponding author.

#### 2.2.2 Feline OA (FeOA) global impression items

Four single-item owner-completed global impression items were developed, in line with regulatory guidance (41–43), as anchor measures to support analysis in the psychometric evaluation study, as described below. The items included in the CD interviews were in pen and paper format; these items were migrated to an eCOA format for the phase 4 field study.

- Global Impression of Cat Quality of Life (OGIC-QoL)—a single item designed to capture owners’ perception of their cat’s overall QoL over the past 7 days on a 4-point response scale (‘Poor’, ‘Fair’, ‘Good’, ‘Very good’).
- Global Impression of Owner Quality of Life (OGIO-QoL)—a single item designed to capture owners’ perception of their own overall QoL over the past 7 days on a 4-point response scale (“Poor’, ‘Fair’, ‘Good’, ‘Very good’)
- Global Impression of Cat Quality of Life Change (OGIC-C)—a single item designed to assess owners’ overall impression of changes in their cat’s QoL over the past 7 days on a 5-point response scale (‘Much better’, ‘Better’, ‘No change’, ‘Worse’, ‘Much worse’).
- Global Impression of Owner Quality of Life Change (OGIO-C)—a single item designed to assess owners’ overall impression of changes in their own QoL over the past 7 days on a five-point response scale (Much better’, ‘Better’, ‘No change’, ‘Worse’, ‘Much worse’).

#### 2.2.3 The VetMetrica™ Feline HRQoL instrument

The owner-completed 20-item VetMetrica™ Feline HRQoL instrument is a validated generic QoL instrument designed to assess the impact of chronic diseases on QoL in cats (20, 21). This instrument comprises three domains (Vitality, Comfort, Emotional wellbeing), assessing both physical and emotional impacts on QoL. While not specific to OA in cats, VetMetrica™ has been validated as an assessment of QoL in cats with OA (22), making it suitable for evaluating the convergent validity of the FeOA-QoL-TS-A. The VetMetrica™ Feline HRQoL instrument was completed by owners in eCOA format in the phase 4 field study.

### 2.3 Stage 1: qualitative concept elicitation (CE) and cognitive debriefing (CD) interviews

#### 2.3.1 Sample and recruitment

A purposive sampling method was used to recruit participants for the interviews, which involved targeting participants based on the eligibility criteria (44). Participants were recruited through veterinarians in the US and UK. The recruiting veterinarians were identified using a network of existing Zoetis contacts. Veterinarians reviewed their databases for cats who met the study eligibility criteria and contacted their owners to invite them to participate. To be eligible, owners had to be at least 18 years of age at the time of recruitment, be an owner of a cat with veterinarian-diagnosed OA in at least one limb, and be verbally fluent and literate in the English language. Veterinarian-diagnosis of OA was confirmed through clinical expertise including detailed medical history, conversations with owner(s), and physical examination. A diagnosis through radiography was not required as the methods above reflect preliminary diagnosis of OA in real-world settings. Further, all the eligible cats recruited for the study were prescribed OA-specific treatment by their veterinarian. Cat and owner demographic characteristics were collected to characterize the sample.

The target sample size was informed by the principle of ‘saturation’, which is considered achieved when no new relevant concepts are identified (i.e., all concepts of importance to participants have been elicited) (45). Previous research suggests saturation can be achieved within 9–17 interviews and that a sample of seven to 10 individuals is appropriate to assess relevance and understanding of measures for a specific concept (46, 47). Additionally, similar qualitative research in cats/dogs have used a sample of 10 owners (48–50). It was anticipated that a minimum sample of eight participants [combined with the existing findings of the systematic literature review and resulting conceptual model (2)] would be sufficient to achieve saturation, while ensuring study feasibility and adequate representation of demographic and clinical characteristics.

#### 2.3.2 Interview procedure

The CE section of the interviews used broad, open-ended questions to facilitate spontaneous elicitation of concepts regarding the impact of cat OA on cat and owner QoL, to confirm the relevance of the identified literature review concepts and identify missing concepts. Owners were asked to describe signs or behaviors that their cat experienced since developing OA, including any signs or behaviors that were indicators of their cat’s QoL. Owners were also asked how their cat’s OA affected their own QoL. If concepts of interest identified in the literature reviews had not emerged through broad exploration, owners were asked more focused questions to ensure the breadth of concepts associated with the impact of OA on cat and owner QoL had been fully investigated. Owners were also asked questions about their cat’s current OA treatment, to explore Treatment Satisfaction.

For the CD section, owners were asked to complete the FeOA-QoL-TS-A using a ‘think aloud’ approach in which they were asked to share their thoughts aloud as they read each instruction/item and chose their response. Owners were then asked detailed questions about their interpretation and understanding of the instruction/item wording, relevance and comprehensiveness of the concepts assessed, and appropriateness of the response options and recall period in the context of use, to generate evidence of content validity. CD was completed following CE to minimize bias in the topics participants reported. Owners were also asked to complete the four global impression items using the same approach. The interviews were conducted in two rounds to allow for instrument refinements between rounds.

#### 2.3.3 Qualitative analysis

All interviews were audio-recorded, via Microsoft Teams, and transcribed verbatim by an accredited transcription vendor. The CE section of the interview transcripts was analyzed in Atlas.Ti software, version 7, using thematic analysis (51). Owner quotes were assigned corresponding concept codes in accordance with a coding scheme agreed amongst the research team. Throughout the data analysis process, the study team met on a regular basis to resolve discrepancies through discussion and consensus-building. The code list was updated iteratively throughout the analysis, and previously coded transcripts were revisited and reviewed to identify instances where the new codes may apply. Codes were applied both deductively (based on prior knowledge) and inductively (as emerging from the data). Saturation analysis was conducted for cat QoL and owner QoL concepts using the CE data to ensure all relevant concepts were elicited. For this, eight transcripts were chronologically grouped into one set of two and two sets of three. Spontaneously reported cat and owner QoL concepts emerging from each set were then iteratively compared. Saturation was considered met if no new concept-relevant information emerged in the final set of interviews (52, 53). The CD section of the transcripts was analyzed in Atlas.Ti using dichotomous codes that were assigned to each instruction, item, response option, and recall period to indicate whether it was understood and relevant. Throughout the coding process, select transcripts were randomly selected for review to ensure consistency.

### 2.4 Stage 2: phase 4 field study

#### 2.4.1 Sample and recruitment

A purposive sampling method was also used to recruit participants for the field study, which involved targeting participants based on the eligibility criteria (54). Owners of cats with OA were recruited by veterinarians from 50 veterinary clinic sites in the UK. To be eligible, participants had to be an owner of a cat aged ≥12 months with veterinarian-confirmed diagnosis of OA who had been prescribed Frunevetmab by a veterinarian. Consistent with Stage 1, veterinarian-diagnosis of OA was confirmed through clinical expertise including detailed medical history, conversations with owner(s), and physical examination. A diagnosis through radiography was not required. Owners of cats who had been prescribed alternative pain medications, such as NSAIDs, opioid analgesics, and systemic corticosteroids, in the previous 2 weeks were not eligible for the study. All breeds and sexes (male/female) were included; however, cats could not be pregnant or lactating or intended for breeding.

A minimum of 90 owners of cats with OA was deemed sufficient to power the planned analyses. This was based on test–retest reliability calculations which determined that a minimum of 45 stable cats would be needed to detect an intraclass correlation coefficient [ICC] of 0.75 (assuming null hypothesis ICC = 0.5, Type I error rate = 0.05, power = 0.80, 50% stable participants by Day 56/63) (55). Previous research suggests that a sample of 50–100 is sufficient for validity analyses (56), further supporting *n* = 90; however, this is dependent on distribution of QoL in the sample. A target of 120 owners of cats with OA allowed for a 25% hypothesized attrition rate, to ensure the recruitment of 90 evaluable cases.

#### 2.4.2 Schedule of assessments

The study comprised six timepoints: Day 0 (Baseline), Day 14 (2 weeks after first dose), Day 28 (second dose), Day 56 (third dose), Day 63 (1 week after third dose), and Day 70 (2 weeks after third dose). All included COA measures (FeOA-QoL-TS-A, VetMetrica™ Feline HRQoL, OGIC-QoL, OGIO-QoL, OGIC-C, and OGIO-C) were completed at all six timepoints on an eCOA device. All COA measures were administered electronically via a study-specific application downloaded on the participants’ own smartphone device. The app also recorded the sex, age, and weight of the cats. The methods of this field study have been summarized in a separate publication (23).

#### 2.4.3 Psychometric analysis

An analysis plan was written prior to the conduct of any analyses. Analyses were conducted in two phases, described in Table 1. Phase A aimed to develop the item-scale structure of the FeOA-QoL-TS-A that would be taken forward to Phase B, including consideration of deletion of poorly performing or redundant items. This was determined based on item response distributions, inter-item correlations, multi-trait analysis, confirmatory factor analysis (CFA), earlier qualitative findings, and the clinical relevance and importance of items. Phase B analyses (internal consistency reliability, test–retest reliability, convergent validity and cross-construct correlations, known groups comparisons, and ability to detect change over time) evaluated the psychometric properties of the resulting item-scale structure. Responder definitions for meaningful change thresholds were also estimated using anchor- and distribution-based methods. All analyses used SAS version 9.4 (57), apart from CFA which was performed using Mplus version 7.3.1 run in R version 4.2.2 and calculation of coefficient omega using R version 4.2.2 (58).

**Table 1:** Summary of psychometric analyses.

Analysis	Description
Phase A: Development of the item-scale structure of the FeOA-QoL-TS-A (Item-level and dimensionality analyses)	
Item response distributions	Item response distributions (frequency and percentage of cat owners selecting each response) were assessed for all timepoints, and items were considered further when a proportion of >30% of respondents selected response options representing the best health state/highest treatment satisfaction (ceiling effect) or worst health state/lowest treatment satisfaction (floor effect).
Inter-item correlations	Correlations between items (inter-item) assessed the homogeneity of items per domain and items correlating very highly (>0.90) were flagged for potential redundancy. Inter-item correlations were assessed using data from Day 28.
Multi-trait analysis	Multi-trait analysis examined whether items in the same domain were correlated adequately. Items were expected to correlate more highly (≥0.40) with items in their own domain, demonstrating item-convergent validity (72). Multi-trait analysis was assessed using data from Day 28.
Confirmatory factor analysis (CFA)	CFA using Day 28 data evaluated the hypothesized three domain conceptual framework. Model fit was assessed using the Comparative Fit Index (CFI), Tucker–Lewis Index (TLI), root mean square error approximation (RMSEA), and weighted root mean square residual (WRMR); CFI and TLI ≥ 0.95 (73), RMSEA <0.10 (74), and WRMR ≤1.0 (75), were considered a good model fit.
Phase B: Evaluation of psychometric properties of resulting item-scale structure (reliability and validity)	
Internal consistency reliability	Internal consistency, concerns the homogeneity of items in a score, and was assessed using Cronbach’s alpha coefficient (≥0.70 for good internal consistency) (76) and omega coefficient values using omega (total) (77). Internal consistency was assessed using data from Day 28.
Test–retest reliability	Test–retest reliability evaluated consistency in scores between Day 56 and Day 70 in a subset of stable cat owners, using intra-class correlation coefficients (ICCs; >0.75 for good test–retest reliability) (78, 79). Cat owners who reported ‘no change’ on the OGIC-QoL (cat QoL) and OGIC-QoL (owner QoL), were defined as ‘stable’ and included in the analysis for the cat QoL and owner QoL domain, respectively. It was assumed that Treatment Satisfaction scores would remain stable between these timepoints; thus, all cat-owner pairs were included in the analysis for the Treatment Satisfaction domain.
Convergent validity and cross construct correlations	Convergent validity was evaluated using data collected at Day 28, by examining the correlations of scores of the VetMetrica Feline HRQoL (all three domains; with Cat QoL domain) and anchor measures [OGIC-QoL (with Cat QoL domain) and OGIO-QoL (with Owner QoL domain)]. Cross-construct correlations were also evaluated by examining correlations of the Cat QoL and Owner QoL domains with the owner and cat measures, respectively. Scores assessing similar or related concepts were expected to have moderate (≥0.30) to strong correlations (≥0.50), whereas scores assessing less closely related concepts were expected to show small (<0.30) or negligible correlations.
Known groups comparisons	Known groups comparisons at Day 28 evaluated differences in scores between cat-owner pairs who differ on variables hypothesized to influence the construct of interest. Groups were defined by responses to the OGIC-QoL or OGIO-QoL (categories: ‘Poor/Fair’, ‘Good’, ‘Very Good’). The magnitude of the differences was considered using between-group effect size (ES) estimates [small difference (ES = 0.20), moderate difference (ES = 0.50), large difference (ES = 0.80)] (80). *F*-test calculated by one-way ANOVAs (comparison of more than two groups) were used to evaluate if differences were statistically significant (*p* ≤ 0.05).
Ability to detect change	Ability to detect change assesses whether scores change over time in line with true change in the construct it measures. Between and within-groups mean change scores were compared from Baseline to Day 56 for the OGIC-QOL and OGIO-QoL (categories: improved = ≥1-catgegory improvement, stable = no change, worsened = ≥1-category worsening) and for the OGIC-C and OGIO-C (categories: improved = ‘Better/Much better’, stable = ‘No change’, worsened = ‘Worse/Much worse’). The magnitude of difference was considered using within-group effect size estimates [small difference (ES = 0.20), moderate difference (ES = 0.50), large difference (ES = 0.80)] (80). *F*-test calculated by one-way ANOVA was used to evaluate if between-group differences were statistically significant (*p* ≤ 0.05).
Deriving meaningful change threshold	Meaningful change thresholds characterize how meaning is attributed to observed changes and differences in scores, beyond what is provided for by statistically significant results. Anchor-based methods were of primary interest with distribution-based methods used as supportive. In anchor-based methods, a patient-centered external indicator is used to identify respondents (cat-owner pairs) who have experienced change on the concept being measured (OA-related QoL). Anchor-based thresholds included within-group responder definitions and between-group minimal important differences in mean change scores (MID) estimated using the OGIC-QoL, OGIO-QoL, OGIC-C, OGIO-C as anchors. Anchor-based change scores had to correlate >0.30 with the domain change score to be considered relevant for interpretation (81). Thresholds were calculated by evaluating within- and between-group mean change scores. The within-group responder definition estimates were calculated using mean change scores for the ‘1-category improved’ group for each anchor from Baseline to Day 56. The between-group MID was defined as the difference between the mean change scores from Baseline to Day 56 for the ‘Minimally improved’ and ‘Stable’ groups for each anchor. A correlation weighted average was calculated using within-group responder definition estimates with Fisher’s *Z* transformation to produce a single value threshold (61). Empirical cumulative distribution function (eCDF) plots were produced to evaluate the performance of the responder definitions produced. Distribution-based estimates of ½ standard deviation (SD) at Baseline and the standard error of measurement (SEM) at Baseline were calculated to identify the amount of change exceeding measurement error.

## 3 Results

### 3.1 Stage 1: qualitative interview results

#### 3.1.1 Sample characteristics

The target sample (*N* = 8) was achieved. A total of eight owners [mean age (range): 59.8 (50–71) years] of cats with a veterinarian-confirmed diagnosis of OA, based in the US (*n* = 4) and UK (*n* = 4), participated in Stage 1 interviews. Most owners were female (*n* = 6) and were educated to a high level (i.e., undergraduate degree or higher; *n* = 7). A range of work status and living locations were reported. On average, participants had been cat owners for 35.8 years and had owned their cat with OA for 11.7 years.

All eight cats were senior (>10 years) (59) with a mean age of 15.2 years (range: 12.0–17.5 years). Most cats were male (*n* = 7) and of mixed breed (*n* = 5). The sample included cats with both indoor only and outdoor access, as well as a range of OA severities. See Supplementary Table 1 for cat characteristic information.

#### 3.1.2 Concept elicitation (CE) results

##### 3.1.2.1 Cat and owner Quality of Life (QoL)

Owners reported a total of 34 cat QoL concepts associated with cat OA. These were categorized into nine core domains: mobility, physical appearance, temperament, vocalizations, energy, sleep, toileting, roaming behaviors, and feeding habits. The most frequently reported impacts related to mobility (*n* = 7), including difficulty jumping up and slowness/stiffness (*n* = 6, each).

In terms of their own QoL, owners reported a total of 19 owner QoL concepts associated with cat OA. These were categorized into seven core domains: impacts on emotional wellbeing, financial cost, activities of daily living (ADL), social functioning, physical functioning, sleep, and environmental adaptations. Impacts on emotional wellbeing (*n* = 6; e.g., feeling sad, worry) and financial costs (*n* = 6; e.g., treatment costs) were most frequently reported. Three owners described how their physical functioning (e.g., due to cat requiring help to access areas) and sleep (e.g., reduced sleep) had been impacted.

##### 3.1.2.2 Treatment Satisfaction

Owners discussed their experience with their cat’s current OA treatment and indicated which aspects impact their level of satisfaction with the treatment. Treatment satisfaction was grouped into six domains: treatment efficacy, cost of treatment, mode of administration, frequency of administration, ease of fitting treatment into daily life, and availability of treatment options. Owners indicated that they would be satisfied with a treatment that is effective at treating signs of cat OA, without side effects, easy to administer, administered less frequently relative to other available options, administered orally or via transdermal application, or incorporates the use of massage or acupuncture.

##### 3.1.2.3 Updated conceptual model and saturation

**Figure 2:**
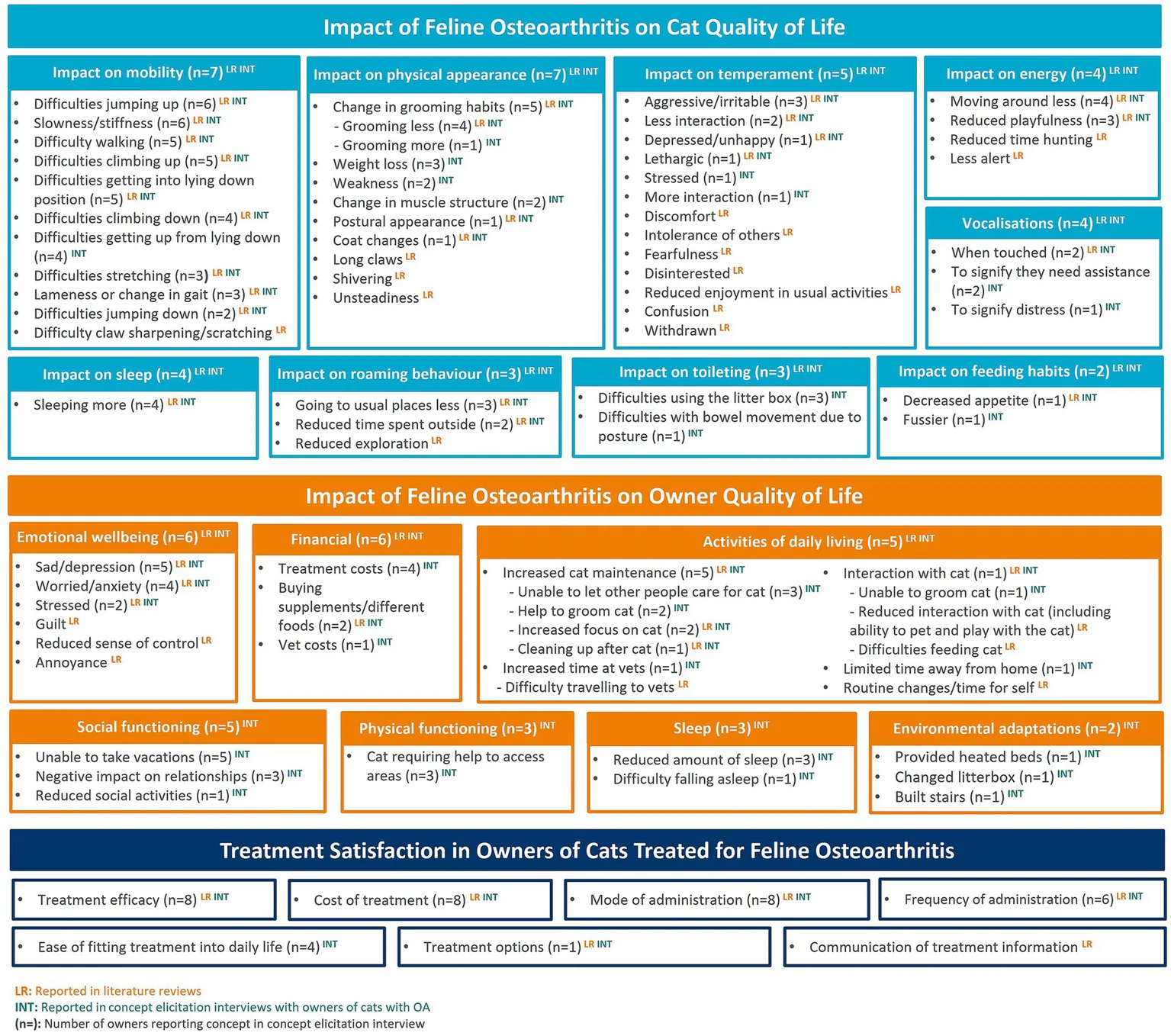
Updated conceptual model of the impact of feline OA on cat and owner QoL and treatment satisfaction.

The conceptual model, developed following earlier literature review findings, was updated to include the CE data from the current study (Figure 2). Most spontaneously reported cat QoL concepts were elicited in the first two sets of interviews (*n* = 25/28 concepts, 89.3%), except for ‘postural changes’, having ‘bowel movement difficulties’ and being ‘aggressive/irritable’. Saturation analysis highlighted that no new owner QoL concepts were identified in the last set of interviews.

#### 3.1.3 Cognitive debriefing (CD) results

##### 3.1.3.1 Round 1

In round one, four cat owners (*n* = 2 US, *n* = 2 UK) completed and debriefed the draft 29-item FeOA-QoL-TS-A and the FeOA global impression items. All instructions and items were well understood by at least three participants (i.e., owners’ description of the item concept matched the intended meaning, and they were able to select a response option that matched their experience). Most items (25/29) were considered relevant to two or more participants (i.e., owners observed or experienced the concept assessed by the item). The most relevant items included: the cat having difficulty ‘walking’ or ‘jumping up’, or ‘sleeping a lot’; the owner being ‘able to interact’ with their cat, having to ‘clean up’ after their cat, feeling ‘sad’, or feeling ‘worried’; and owner satisfaction with treatment overall, treatment frequency, or treatment administration. Less relevant items included the cat appearing ‘happy’, the cat being ‘around the owner and those they live with’, the owner having to ‘travel to the vets’, and owner satisfaction with ‘fitting treatment into daily life’. Two participants understood the response options and indicated they were appropriate; however, the other two participants selected inappropriate response options due to issues switching between positive and negative directionality of the response scale across items. All participants understood the recall period and two indicated that it was appropriate.

All FeOA global impression items were understood by all participants, except for the OGIC-C due to issues with the hypothetical phrasing. All participants asked understood the response options, with varied feedback on appropriateness.

##### 3.1.3.2 Updates following round 1

Based on the round 1 findings, modifications were made to the items (summarized in Supplementary Figure 1), recall period, and response scale of the draft FeOA-QoL-TS-A:

- Removal of recall period (‘past 7 days’) from the Treatment Satisfaction domain due to feedback that it was not needed or too short to capture treatment efficacy. The response options in this domain were revised to reflect this change;
- The addition of a ‘not applicable’ response option (‘I have not seen my cat do this’) for behaviors not observed by owners (‘toileting’ item in Cat QoL domain);
- Updating the items to be consistently negatively framed to improve consistency with the directionality of the response scale used.

##### 3.1.3.3 Round 2

In round 2, four cat owners (*n* = 2 US, *n* = 2 UK) completed and debriefed the updated draft 29-item FeOA-QoL-TS-A and FeOA global impression items. All instructions and items were well understood by at least three participants. Over half of the items (15/29) were considered relevant to at least two participants. The most relevant items included: the cat having difficulty ‘walking’, ‘moving slowly’, ‘jumping up/down’, ‘lying down/getting up’, or being ‘active’; the owner feeling ‘worried’; and owner satisfaction with treatment administration, financial cost, or treatment overall. Items that were not relevant included items assessing cat’s ‘appetite’, owners’ ‘having to travel to the vets’ and owner satisfaction with ‘fitting treatment into daily life’. All participants understood the response options and considered them appropriate. All participants understood the recall period; however, three participants indicated that it was not appropriate as it was too short to experience item concepts or detect changes in response to treatment.

All participants understood the FeOA global impression items and response options. For the OGIC-QoL and OGIO-QoL, three participants reported that the response option ‘Excellent’ was not appropriate; this was removed in line with feedback from all rounds. For the OGIC-C and OGIO-C, two participants reported no difference between the options ‘Worse’ and ‘Much worse’; these were retained to align with regulatory guidance.

**Figure 3:**
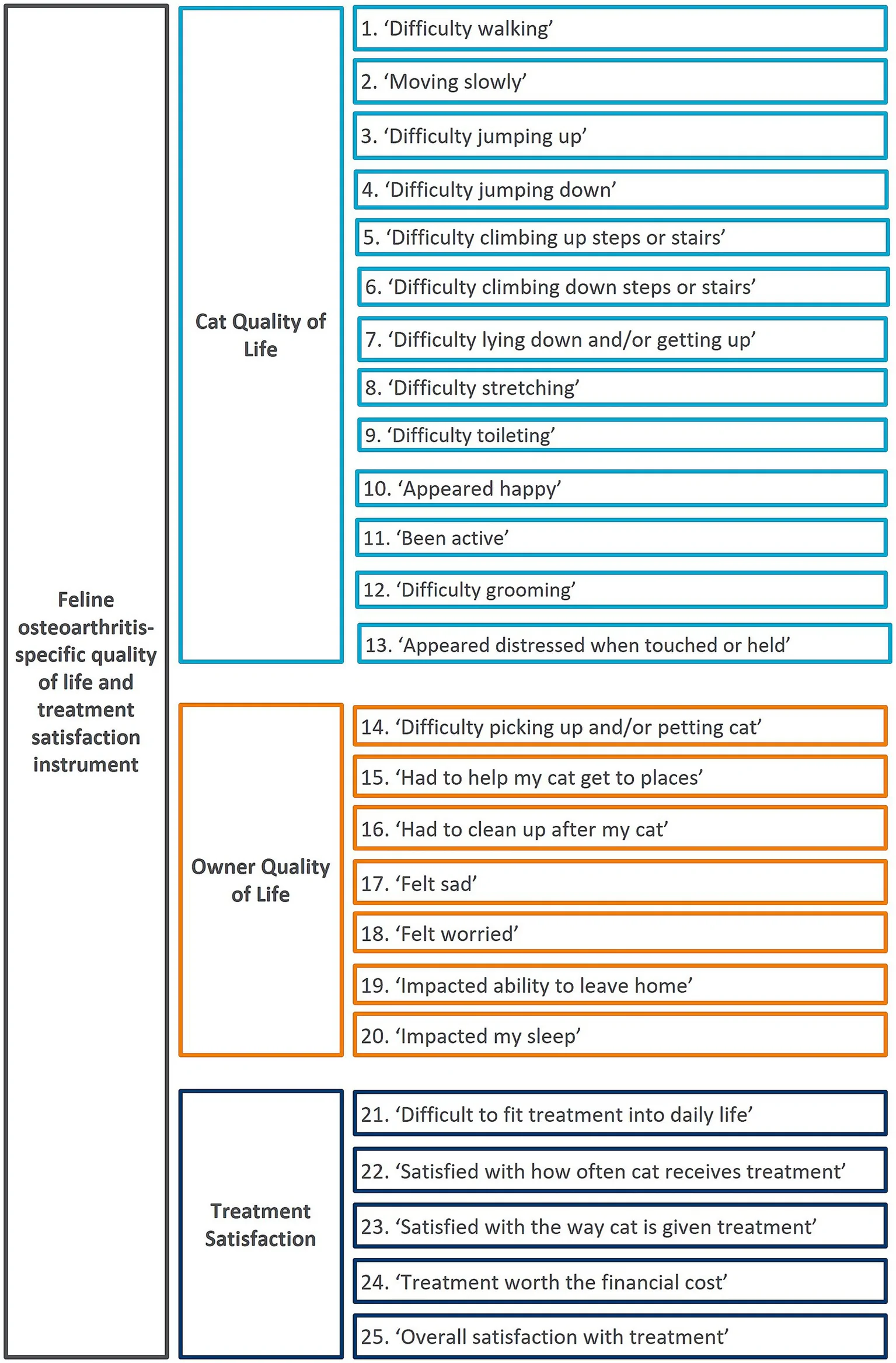
Updated conceptual framework of the FeOA-QoL-TS-A, following conduct of qualitative interviews.

Based on the round 2 findings, modifications were made to the items (summarized in Supplementary Figure 1) of the draft FeOA-QoL-TS-A. The ‘not applicable’ response option (‘I have not seen my cat do this’) was added to two Cat QoL domain items assessing climbing up/down steps or stairs. This resulted in a 25-item instrument taken forward for the phase 4 field study and psychometric evaluation (Figure 3).

### 3.2 Stage 2: psychometric evaluation study results

#### 3.2.1 Sample characteristics

The target sample was achieved (*N* = 120, *n* = 90 evaluable cases). A total of 139 cat owners were enrolled in the phase 4 field study at Baseline (Day 0); 94 of whom (67.6%) completed the FeOA-QoL-TS-A at all six timepoints. Owner demographics were not collected as part of the validation study as they were not considered to be pertinent to the study objectives. The feline sample included an equal proportion of female (*n* = 70; 50.4%) and male (*n* = 69; 49.6%) cats. Most of the sample comprised neutered male cats (*n* = 59, 42.4%) and neutered female cats (*n* = 54, 38.8%), followed by female unneutered (*n* = 16, 11.5%) and male unneutered (*n* = 10, 7.2%) cats. The sample comprised senior cats with a mean age of 13.6 years (range: 5.0–20.0 years), and the mean cat weight was 4.5 kg, in line with average weight of adult domestic cats (60). See Supplementary Table 1 for details.

#### 3.2.2 Phase A: development of the item-scale structure of the FeOA-QoL-TS-A

##### 3.2.2.1 Quality of completion

Form level quality of completion indicated a final attrition rate of 32.4% by Day 70, which was higher than the 25% attrition rate hypothesized in the analysis plan. Due to electronic administration of the instrument prohibiting respondents to skip items, item level quality of completion was not assessed.

##### 3.2.2.2 Item response distributions

Item response distributions were calculated at all timepoints. Item-level response distributions, as assessed at Baseline, demonstrated a good spread of responses across the response scale for most items, except for Item 9 (‘difficulty toileting’), Item 16 (‘clean up after cat’), Item 19 (‘impacted ability to leave home’), Item 20 (‘impacted sleep’), Item 21 (‘fitting treatment into daily life’), Item 22 (‘satisfaction with treatment frequency’), Item 23 (‘satisfaction with treatment administration’), Item 24 (‘financial cost of treatment’), and Item 25 (‘overall treatment satisfaction’). Across all Cat QoL and Owner QoL domain items, there was a higher proportion of respondents selecting responses indicative of better health states at later timepoints, resulting in a skewed spread of responses; however, this was expected due to known effectiveness of Frunevetmab (23). For the treatment satisfaction domain items, there was little change in the spread of responses over time, which may reflect consistency of treatment.

Floor and ceiling effects were considered present in an item when at least 30% of respondents selected the worst health state/lowest treatment satisfaction (floor) or best health state/highest treatment satisfaction (ceiling) on the response scale. No floor effects were observed at any timepoints except for Item 11 at Baseline (‘been active’, 30.9%). A ceiling effect was identified at Baseline for five Cat QoL domain items including: Item 7 (‘difficulty lying down/getting up from lying down’, 31.7%), Item 8 (‘difficulty stretching’, 41.7%), Item 9 (‘difficulty toileting’, 52.5%), Item 12 (‘difficulty grooming’, 30.9%), and Item 13 (‘appeared distressed when touched or held’, 43.9%). A ceiling effect was also identified at Baseline for five Owner QoL domain items including: Item 14 (‘difficulty picking up and/or petting cat’, 50.4%), Item 15 (‘help cat get to places’, 46.8%), Item 16 (‘clean up after cat’, 68.3%), Item 19 (‘impacted ability to leave home’, 73.4%), and Item 20 (‘impacted sleep’, 64.0%). In the Treatment Satisfaction domain, between 45.3 and 86.3% of respondents reported the highest Treatment Satisfaction domain score possible on each item (ceiling) at Baseline. Ceiling effects were also observed at later timepoints across items, as expected due to the efficacy of Frunevetmab as a treatment (23).

##### 3.2.2.3 Inter-item correlations

Inter-item correlations were calculated using data from Day 28 to provide an initial exploration of dimensionality. Items generally correlated more strongly with items within their own hypothesized domains than between domains (Table 2). Very strong inter-item correlations (*r* = 0.90) between four item pairs were observed, indicating potential item redundancy, specifically between: Cat QoL domain Item 3 (‘difficulty jumping up’) and Item 4 (‘difficulty jumping down’); Cat QoL domain Item 5 (‘difficulty climbing up steps or stairs’) and Item 6 (‘difficulty climbing down steps or stairs’); Owner QoL domain Item 17 (‘sad’) and Item 18 (‘worried’); and Owner QoL domain Item 19 (‘impacted ability to leave home’) and Treatment Satisfaction domain Item 21 (‘fitting treatment into daily life’). Weak correlations (<0.40) were observed for Cat QoL domain Item 9 (‘difficulty toileting’) and Item 13 (‘appeared distressed when touched or held’) with most other items in their hypothesized domain.

**Table 2:** Inter-item correlation matrix at day 28 (*n* = 113).

Item	1	2	3	4	5	6	7	8	9	10	11	12	13	14	15	16	17	18	19	20	21	22	23	24	25
1. Difficulty walking	1.00	–	–	–	–	–	–	–	–	–	–	–	–	–	–	–	–	–	–	–	–	–	–	–	–
2. Moving slowly	0.85	1.00	–	–	–	–	–	–	–	–	–	–	–	–	–	–	–	–	–	–	–	–	–	–	–
3. Difficulty jumping up	0.66	0.65	1.00	–	–	–	–	–	–	–	–	–	–	–	–	–	–	–	–	–	–	–	–	–	–
4. Difficulty jumping down	0.65	0.59	0.91	1.00	–	–	–	–	–	–	–	–	–	–	–	–	–	–	–	–	–	–	–	–	–
5. Difficulty climbing up	0.74	0.77	0.80	0.73	1.00	–	–	–	–	–	–	–	–	–	–	–	–	–	–	–	–	–	–	–	–
6. Difficulty climbing down	0.73	0.77	0.69	0.72	0.91	1.00	–	–	–	–	–	–	–	–	–	–	–	–	–	–	–	–	–	–	–
7. Difficulty lying down/getting up	0.72	0.71	0.68	0.69	0.72	0.71	1.00	–	–	–	–	–	–	–	–	–	–	–	–	–	–	–	–	–	–
8. Difficulty stretching	0.56	0.65	0.53	0.57	0.53	0.53	0.83	1.00	–	–	–	–	–	–	–	–	–	–	–	–	–	–	–	–	–
9. Difficulty toileting	0.29	0.26	0.29	0.24	0.19	0.31	0.23	0.28	1.00	–	–	–	–	–	–	–	–	–	–	–	–	–	–	–	–
10. Happy	0.43	0.55	0.42	0.42	0.49	0.44	0.45	0.53	0.27	1.00	–	–	–	–	–	–	–	–	–	–	–	–	–	–	–
11. Active	0.50	0.65	0.49	0.50	0.53	0.50	0.58	0.48	0.26	0.67	1.00	–	–	–	–	–	–	–	–	–	–	–	–	–	–
12. Difficulty grooming	0.26	0.37	0.43	0.46	0.33	0.33	0.43	0.46	0.36	0.39	0.42	1.00	–	–	–	–	–	–	–	–	–	–	–	–	–
13. Distressed when touched/ held	0.20	0.30	0.26	0.30	0.30	0.26	0.34	0.20	−0.03	0.46	0.42	0.40	1.00	–	–	–	–	–	–	–	–	–	–	–	–
14. Difficulty picking up/ petting cat	0.52	0.52	0.47	0.48	0.47	0.46	0.60	0.50	0.06	0.58	0.42	0.37	0.82	1.00	–	–	–	–	–	–	–	–	–	–	–
15. Help cat get to places	0.65	0.59	0.73	0.70	0.59	0.58	0.54	0.38	0.39	0.45	0.45	0.37	0.18	0.44	1.00	–	–	–	–	–	–	–	–	–	–
16. Clean up after cat	0.27	0.37	0.33	0.30	0.35	0.36	0.23	0.44	0.75	0.40	0.36	0.42	0.12	0.17	0.35	1.00	–	–	–	–	–	–	–	–	–
17. Sad	0.64	0.65	0.63	0.66	0.68	0.71	0.74	0.64	0.42	0.57	0.58	0.49	0.26	0.53	0.53	0.42	1.00	–	–	–	–	–	–	–	–
18. Worried	0.68	0.67	0.61	0.59	0.67	0.71	0.70	0.63	0.41	0.52	0.50	0.49	0.21	0.53	0.51	0.32	0.95	1.00	–	–	–	–	–	–	–
19. Ability to leave home	0.47	0.43	0.64	0.73	0.61	0.63	0.69	0.70	0.29	0.32	0.39	0.34	0.01	0.48	0.67	0.23	0.74	0.72	1.00	–	–	–	–	–	–
20. Sleep	0.50	0.52	0.64	0.67	0.64	0.56	0.81	0.67	0.36	0.43	0.49	0.52	0.25	0.40	0.49	0.18	0.73	0.71	0.74	1.00	–	–	–	–	–
21. Fitting treatment into daily life	0.13	0.09	0.12	0.04	0.14	0.15	0.26	0.35	−0.14	0.02	0.01	0.14	−0.06	−0.14	−0.04	−0.05	0.06	0.20	0.96	0.30	1.00	–	–	–	–
22. Frequency of treatment	−0.17	−0.16	−0.16	−0.23	−0.18	−0.25	−0.17	−0.10	−0.25	−0.18	−0.28	−0.10	−0.15	−0.19	−0.24	−0.19	−0.29	−0.32	−0.08	−0.13	0.59	1.00	–	–	–
23. Way treatment received	−0.14	−0.13	−0.17	−0.22	−0.17	−0.16	−0.04	−0.05	−0.07	−0.27	−0.18	−0.09	−0.30	−0.30	−0.15	−0.21	−0.21	−0.23	0.05	−0.03	0.52	0.83	1.00	–	–
24. Financial cost of treatment	−0.19	−0.25	−0.22	−0.28	−0.20	−0.20	−0.11	−0.13	−0.06	−0.19	−0.37	−0.24	−0.35	−0.11	−0.26	−0.18	−0.20	−0.13	0.07	0.06	0.50	0.67	0.64	1.00	–
25. Overall treatment satisfaction	−0.42	−0.47	−0.39	−0.42	−0.38	−0.38	−0.40	−0.39	−0.25	−0.53	−0.52	−0.32	−0.46	−0.38	−0.36	−0.11	−0.42	−0.43	0.04	−0.42	0.14	0.73	0.68	0.73	1.00

##### 3.2.2.4 Dimensionality of the FeOA-QoL-TS-A

The appropriateness of grouping the items into three hypothesized multi-item scores (comprising 25 items) was tested using multi-trait analysis and CFA. Multi-trait analysis was conducted using data from Day 28 and involved correlating individual items with each domain score; items were expected to have stronger correlations with their hypothesized domain score.

Most items correlated at least moderately (>0.40) with their hypothesized domain, except Cat QoL domain Item 9 (‘difficulty toileting’; *r* = 0.33), Cat QoL domain Item 13 (‘appeared distressed when touched or held’; *r* = 0.35) and Owner QoL domain Item 16 (‘clean up after cat’; *r* = 0.31), indicating that these items could be candidates for removal. Most items correlated stronger with their hypothesized domain, except for one Cat QoL domain item which correlated more strongly with the Owner QoL domain [Item 9 (‘difficulty toileting’); Cat QoL = 0.33, Owner QoL = 0.50]. In addition, five Owner QoL domain items correlated more strongly with the Cat QoL domain; including Item 14 (‘difficulty picking up and/or petting cat’; Owner QoL = 0.40, Cat QoL = 0.60), Item 15 (‘help cat get to places’; Owner QoL = 0.51, Cat QoL = 0.65), Item 16 (‘clean up after cat’; Owner QoL = 0.31, Cat QoL = 0.46), and Item 20 (‘impacted sleep’; Owner QoL = 0.58, Cat QoL = 0.67). These findings highlighted the need to further inspect these items.

CFA was conducted using data from Day 28 to assess the fit of the hypothesized model to the data. Standardized factor loadings of items on their hypothesized domains all exceeded 0.40 except for Item 21 (‘fitting treatment into daily life’), which loaded on the Treatment Satisfaction domain (0.34). The Cat and Owner QoL domains were strongly related (0.86); however, both correlated negatively with the Treatment Satisfaction domain (−0.39 and −0.30, respectively), as scoring is interpreted in the opposite direction for this response scale. Fit statistics for this model were not considered acceptable [Comparative Fit Index (CFI) = 0.949; Tucker–Lewis Index (TLI) = 0.943; root mean square error of approximation (RMSEA) = 0.121; weighted root mean square residual (WRMR) = 1.554].

##### 3.2.2.5 Item reduction

Item reduction was informed by CFA, performance across other psychometric analyses, and qualitative findings:

- Item 21 (‘fitting treatment into daily life’) had a poor factor loading in the first CFA model, suggesting that this item did not fit with other Treatment Satisfaction domain items and had potential redundancy with Item 19 (‘impacted ability to leave home’).
- Inter-item correlations suggested potential redundancy between two item pairs in the Cat QoL domain [Item 3 (‘difficulty jumping up’) and Item 4 (‘difficulty jumping down’); and Item 5 (‘difficulty climbing up steps or stairs’) and Item 6 (‘difficulty climbing down steps or stairs’)], due to very strong inter-item correlations (*r* ≥ 0.90). The items were also considered conceptually similar.
- Cat QoL domain Item 13 (‘appeared distressed when touched or held’) had a weak correlation with its own hypothesized domain (multi-trait analysis). This item also correlated strongly with Owner QoL domain Item 14 (‘difficulty picking up/and or petting cat’) and was considered conceptually similar.
- Owner QoL domain Item 16 (‘clean up after cat’) had a weak correlation with its own hypothesized domain and correlated more strongly with the Cat QoL domain, indicating that the underlying concept of toileting issues may be better assessed by Cat QoL domain Item 9 (‘difficulty toileting’).

**Figure 4:**
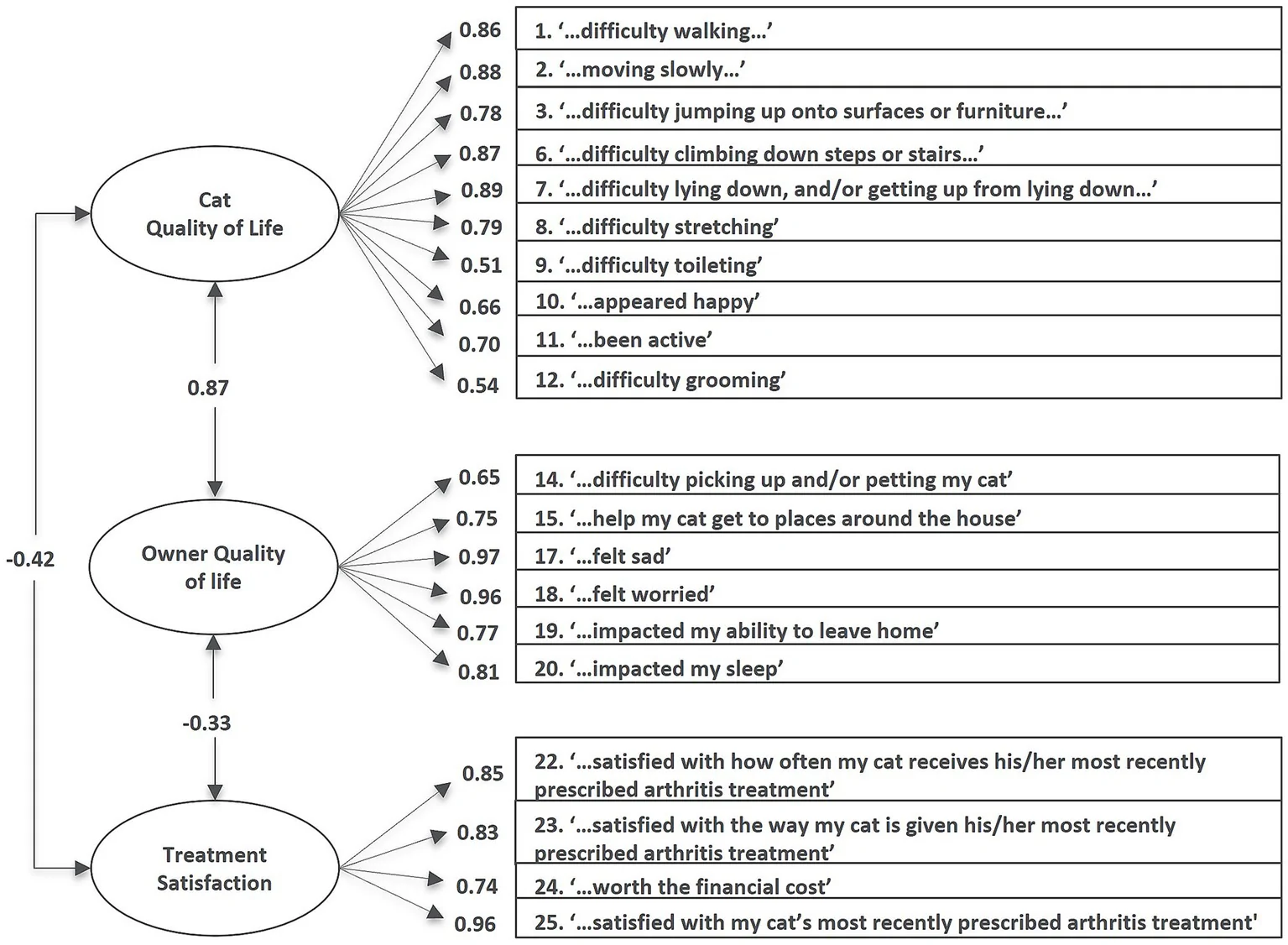
Confirmatory factor analysis factor loadings of the final FeOA-QoL-TS-A at day 28 (*n* = 113).

Repeating the CFA without Item 4 (‘difficulty jumping up’), Item 5 (‘difficulty climbing up steps or stairs’), Item 13 (‘appeared distressed when touched or held’), Item 16 (‘clean up after cat’), and Item 21 (‘fitting treatment into daily life’) saw an improvement in fit statistics (CFI = 0.977; TLI = 0.974; RMSEA = 0.077; WRMR = 0.988, Figure 4), suggesting that this revised structure was acceptable. Almost all items, except Item 9 (‘difficulty toileting’), correlated well (>0.40; Table 3) with their domain score, supporting item-convergent validity through multi-trait analysis. Most items correlated higher with their respective domains, except for Item 9 (‘difficulty toileting’; Cat QoL domain), Item 14 (‘difficulty picking up and/or petting cat’; Owner QoL domain), Item 15 (‘help cat get to places’; Owner QoL domain), and Item 20 (‘impacted sleep’; Owner QoL domain). These items all had stronger correlations with the other QoL domain (Cat or Owner); however, all Owner QoL items still correlated well with their own domain (>0.40). Given the high correlations between the Cat and Owner QoL domains, and overall item performance, no further changes were made to the Owner QoL items. Item 9 was retained despite item performance due to improved fit statistics in the CFA model when included. The item removal decisions are summarized in Supplementary Figure 1.

**Table 3:** Multi-trait analysis of the items in each hypothesized domain at day 28 (*n* = 113).

Scale	Item	Polyserial item-scale correlations		
		Cat QoL	Owner QoL	Treatment satisfaction
Cat QoL	1. Difficulty walking	0.743	0.630	−0.214
	2. Moving slowly	0.785	0.628	−0.239
	3. Difficulty jumping up	0.752	0.609	−0.204
	4. Difficulty jumping down	0.755	0.618	−0.262
	5. Difficulty climbing up	0.785	0.665	−0.206
	6. Difficulty climbing down	0.751	0.651	−0.222
	7. Difficulty lying down/getting up	0.777	0.701	−0.163
	8. Difficulty stretching	0.681	0.647	−0.151
	9. Difficulty toileting	0.330	0.496	−0.155
	10. Happy*	0.578	0.519	−0.288
	11. Active*	0.657	0.560	−0.329
	12. Difficulty grooming	0.482	0.455	−0.169
	13. Distressed when touched/held	0.353	0.278	−0.327
Owner QoL	14. Difficulty picking up/petting cat	0.595	0.404	−0.251
	15. Help cat get to places	0.650	0.513	−0.239
	16. Clean up after cat	0.464	0.305	−0.179
	17. Sad	0.758	0.793	−0.253
	18. Worried	0.745	0.840	−0.232
	19. Ability to leave home	0.633	0.626	0.048
	20. Sleep	0.671	0.579	−0.110
Treatment Satisfaction	21. Fitting treatment into daily life*	0.143	0.094	0.443
	22. Frequency of treatment	−0.228	−0.231	0.768
	23. Way treatment received	−0.189	−0.165	0.736
	24. Financial cost of treatment	−0.273	−0.132	0.672
	25. Overall Treatment Satisfaction	−0.499	−0.333	0.656

Correlation coefficient between each item and its own corrected domain should be at least 0.40. ^*^Symbol means that the item was reverse scored. QoL, Quality of Life.

Following item removal, the structure of the resulting 20-item FeOA-QoL-TS-A was confirmed as three separate domain scores (see Figure 4): Cat QoL (10 items), Owner QoL (6 items), and Treatment Satisfaction (4 items) domains. The items within each domain are used to calculate the respective domain score. There is no total score across the three domains.

#### 3.2.3 Phase B: evaluation of the psychometric properties of the resulting item-scale structure

##### 3.2.3.1 Internal consistency

Internal consistency reliability of the Cat QoL, Owner QoL, and Treatment Satisfaction domains was examined using data from Day 28. Cronbach’s alpha values were well above the *a priori* threshold of ≥0.70 for all three domain scores of the FeOA-QoL-TS-A (Cat QoL = 0.88, Owner QoL = 0.84, and Treatment Satisfaction = 0.84), providing evidence for internally consistent (homogenous) scales. Omega coefficients values were also calculated at Day 28 using omega (total) and demonstrated good reliability >0.70 (Cat QoL = 0.90, Owner QoL = 0.85, Treatment Satisfaction = 0.84) for all three domain scores.

##### 3.2.3.2 Test–retest reliability

Stable cats were defined as those cats with no change in score between Days 56 and 70 on the OGIC-QoL; 60 cats met this criterion. Similarly, stable cat owners were defined as those owners with no change in score between Days 56 and 70 on the OGIO-QoL; 69 owners met this criterion. For the Treatment Satisfaction domain, it was assumed scores would remain stable throughout the study as treatment did not change; therefore, all cat owners with Day 56 to Day 70 data were included in the analysis (*N* = 94). Test–retest reliability was good (ICC > 0.75) for the Cat QoL, Owner QoL, and Treatment Satisfaction domain scores (Table 4).

**Table 4:** Test–retest reliability (*n* = 94).

Scale	Test–retest reliability						
	*n*	Reliability (ICC)	95% confidence intervals		Day 56 Mean (SD)	Day 70 Mean (SD)	Pearson correlation coefficient
			Lower	Upper			
Cat QoL	60	0.88	0.81	0.93	0.72 (0.59)	0.68 (0.61)	0.88
Owner QoL	69	0.78	0.67	0.86	0.31 (0.41)	0.32 (0.47)	0.79
Treatment Satisfaction	94	0.77	0.67	0.84	3.32 (0.72)	3.36 (0.72)	0.76

<0.50 = poor reliability; 0.50–0.75 = moderate reliability; 0.75–0.90 = good reliability; >0.90 = excellent reliability. ICC, intraclass correlation coefficient; SD, Standard Deviation; QoL, Quality of Life.

##### 3.2.3.3 Convergent validity and cross-construct correlations

To evaluate convergent validity, correlations of the Cat QoL domain with the VetMetrica™ Feline HRQoL domain scores and OGIC-QoL were evaluated using data collected at Day 28 (Table 5). Similarly, correlations of the Owner QoL domain and the OGIO-QoL were evaluated. Stronger correlations were hypothesized between these measures of similar constructs. The correlations between the Cat QoL domain and all domains of the VetMetrica™ Feline HRQoL (range: −0.70 to −0.80) and the OGIC-QoL (−0.73) were strong, meeting or exceeding the hypothesized level for measures expected to correlate moderately (≥0.30 but <0.50) or strongly (≥0.50) with this domain, supporting convergent validity evidence. The correlation between the Owner QoL domain and the OGIO-QoL was strong (−0.65), meeting the hypothesized level (≥0.50) and supporting convergent validity evidence.

**Table 5:** Convergent validity and cross-construct Pearson correlations at day 28 (*n* = 113).

Instrument/item score	Cat QoL domain	Owner QoL domain
VetMetrica Feline HRQoL-Vitality	−0.77	−0.62
VetMetrica Feline HRQoL-Comfort	−0.80	−0.72
VetMetrica Feline HRQoL-Emotional Wellbeing	−0.70	−0.67
OGIC-QoL	−0.73	−0.69
OGIO-QoL	−0.66	−0.65

Blue indicates convergent correlations. Orange indicates cross-construct correlations. A strong correlation will be defined as ≥0.50, moderate correlation as ≥0.30 but <0.50, and weak correlation as <0.30. QoL, Quality of Life.

Cross-construct correlations of the Cat QoL and Owner QoL domains with the owner and cat measures, respectively, were evaluated to explore the relationship between related but distinct constructs. Weaker correlations were hypothesized between these measures. For the Cat QoL domain, the correlation with the OGIO-QoL was strong (−0.66), exceeding the hypothesized level (<0.30). For the Owner QoL domain, all correlations with the VetMetrica™ Feline HRQoL and the OGIC-QoL were strong (range: −0.62 to −0.72), exceeding the hypothesized level (<0.30).

For the VetMetrica™ Feline HRQoL domain scores and OGIC-QoL, higher correlations were observed for the Cat QoL domain compared to the Owner QoL domain, as predicted. The OGIO-QoL showed a marginally higher correlation with the Cat QoL domain (−0.66) compared with the Owner QoL domain (−0.65), reflecting the close relationship between these two QoL domains identified in the CFA.

##### 3.2.3.4 Known groups comparisons

The Cat QoL domain score was compared among groups who differed in overall QoL as measured by OGIC-QoL response categories. There was a pattern of significantly higher mean scores (indicating worse QoL) for cats who also scored higher (worse) on the OGIC-QoL on Day 28 (*p* < 0.001), with the expected monotonic increases in means across the groups. Large effect sizes between the ‘Poor or Fair’ and ‘Good’ groups (−1.07) and the ‘Poor or Fair’ and ‘Very Good’ groups (−2.49) further provide known groups evidence (Table 6). A pattern of higher scores (indicating worse QoL) was also reported by owners who reported worse scores on the OGIO-QoL, with statistically significant differences between group means for the Owner QoL domain score (*p* < 0.001). Large effect sizes between the ‘Poor or Fair’ and ‘Good’ groups (−1.09) and the ‘Poor or Fair’ and ‘Very Good’ groups (−2.36) further provide known groups evidence (Table 6).

**Table 6:** Known-group comparisons of the Cat QoL and Owner QoL domains (*n* = 113).

Domain	Comparison group	*n*	Mean (SD)	Median	Between group effect size	*p* value
Cat QoL	Poor or Fair	19	1.7 (0.62)	1.60	Ref.	<0.001
	Good	47	1.1 (0.53)	1.00	−1.07	
	Very Good	47	0.5 (0.42)	0.40	−2.49	
Owner QoL	Poor or Fair	12	1.3 (0.93)	1.00	Ref.	<0.001
	Good	48	0.6 (0.50)	0.50	−1.09	
	Very Good	53	0.2 (0.25)	0.00	−2.36	

Magnitude of effect size: small change (ES = 0.20), moderate change (ES = 0.50), and large change (ES = 0.80). SD, Standard Deviation; QoL, Quality of Life.

##### 3.2.3.5 Ability to detect change

Within-group effect sizes and between-groups one-way ANOVA *F*-test were calculated to evaluate the magnitude and significance in change scores between each group (improved, stable, worsened) for the Cat and Owner QoL domains (Table 7).

**Table 7:** Ability to detect change (*n* = 98).

Anchor/domain	Comparison group	*n*	Mean change score (SD)	Median change score	Min, Max	Within group effect size	Between groups *p* value
Cat QoL domain between Baseline and Day 56							
OGIC-QoL	Improved: ≥1 grade improvement	54	−1.3 (0.69)	−1.1	−3.3, 0.0	−1.63	<0.001
	Stable: no change	34	−0.4 (0.53)	−0.5	−1.8, 0.7	−0.56	
	Worsened: ≥1 grade worsening	10	−0.1 (0.92)	−0.1	−1.2, 2.1	−0.11	
OGIC-C	Improved: “Better” or “Much better”	81	−1.0 (0.74)	−0.8	−3.3, 0.5	−1.26	0.001
	Stable: no change	16	−0.2 (0.95)	−0.3	−2.2, 2.1	−0.30	
	Worsened: “Worse” or “Much worse”	1	0.0 (N. A.)	0.0	0.0, 0.0	N. A.	
Owner QoL domain between Baseline and Day 56							
OGIO-QoL	Improved: ≥1 grade improvement	43	−1.1 (0.80)	−1.0	−3.0, 0.0	−1.33	<0.001
	Stable: no change	36	−0.5 (0.42)	−0.5	−1.7, 0.3	−0.95	
	Worsened: ≥1 grade worsening	19	−0.2 (0.70)	−0.3	−1.0, 1.8	−0.38	
OGIO-C	Improved: “Better” or “Much better”	53	−1.0 (0.79)	−0.7	−3.0, 0.0	−1.22	<0.001
	Stable: no change	43	−0.4 (0.56)	−0.5	−1.5, 1.8	−0.82	
	Worsened: “Worse” or “Much worse”	2	0.6 (0.35)	0.6	0.3, 0.8	1.24	

Magnitude of effect size: small change (ES = 0.20), moderate change (ES = 0.50), and large change (ES = 0.80). OGIC-C, Owner Global Impression of Cat Quality of Life Change; OGIC-QoL, Owner Global Impression of Cat Quality of Life; OGIO-C, Owner Global Impression of Owner Quality of Life Change; OGIO-QoL, Global Impression of Owner Quality of Life; SD, Standard Deviation; QoL, Quality of Life.

For the Cat QoL domain score, statistically significant differences were observed in the mean change score between the improved, stable, and worsened groups when defined using the OGIC-QoL (*p* < 0.001) and OGIC-C (*p* = 0.001), from Baseline to Day 56. Within-group effect sizes were larger for the improved group compared with the stable group when defined using the OGIC-QoL and OGIC-C, providing strong evidence of the ability to detect improvement in cat QoL. Given the small sample sizes for the worsened group for the OGIC-QoL and OGIC-C, no effect size could be produced.

Similarly, for the Owner QoL domain score, statistically significant differences were observed in the mean change score between the improved, stable, and worsened groups when defined using the OGIO-QoL (*p* < 0.001) and OGIO-C (*p* < 0.001), from Baseline to Day 56. Within-group effect sizes were larger for the improved group compared with the stable group when defined using the OGIC-QoL and OGIC-C, providing strong evidence of the ability to detect improvement in owner QoL. Due to the small sample sizes for the worsened group for the OGIO-QoL and OGIO-C, a small effect size in the wrong direction for worsening (i.e., negative change score) was observed for the OGIO-QoL and a large effect size was produced for the OGIO-C, providing no clear evidence of the ability to detect change in worsening of owner QoL.

As the Treatment Satisfaction domain had no anchors, a descriptive summary of change for each timepoint from Baseline was produced to descriptively explore the ability to detect change. There were minimal changes from Baseline in mean scores observed across Days 14–70 (0.00–0.10); therefore, it was not possible to determine this domain’s ability to detect changes over time. Given the known clinical effectiveness of the trial treatment (23), stability in Treatment Satisfaction domain score was expected and not a concern.

##### 3.2.3.6 Meaningful change

###### 3.2.3.6.1 Anchor-based methods

Change correlations in Cat QoL and Owner QoL domain scores from Baseline to Day 56 showed that the OGIC-QoL (*r* = 0.73) and OGIC-C (*r* = 0.46) were appropriate for use as anchors for the Cat QoL domain, and the OGIO-QoL (*r* = 0.61) and OGIO-C (*r* = 0.52) were appropriate for use as anchors for the Owner QoL domain, correlating >0.30 (61). The between-group minimal important difference (MID) estimate for the Cat QoL domain was −0.66 for the OGIC-QoL and −0.62 for the OGIC-C anchor. For the Owner QoL domain, the MID estimate was −0.47 for the OGIO-QoL and −0.42 for the OGIO-C anchor. This suggested a between-group MID value of approximately −0.60 would be applicable for the Cat QoL domain and a value of −0.40 would be applicable for the Owner QoL domain.

Calculation of within-group mean change scores for the ‘1-category improved’ group for each anchor provided a range of estimates [−0.85 to −1.09, 95% confidence interval (CI) range: −0.65 to −1.27] for the Cat QoL domain and a similar range (−0.85 to −0.96, 95% CI range: −0.62 to −1.20) for the Owner QoL domain. The findings suggest a responder definition between −0.85 and −1.0 would be applicable for both Cat and Owner QoL domains. Triangulation of these ‘1-category improved’ group estimates provided a single responder definition of −1.0 for the Cat QoL domain and of −0.9 for the Owner QoL domain (rounded to one decimal place for ease of interpretation). Anchor-based evaluation of meaningful change for the Treatment Satisfaction domain was not explored as no suitable anchors were available and, as cats were expected to remain stable after initial dose of treatment, treatment satisfaction concepts were expected to remain stable.

The empirical cumulative distribution function (eCDF) plots are displayed in the supplementary materials and show that each responder definition classifies a low proportion of respondents who are ‘worsened’ or ‘stable’ as improved, while classifying a high proportion (>50%) of ‘minimally improved’ or ‘much improved’ respondents as improved.

###### 3.2.3.6.2 Distribution-based methods

Distribution-based estimates of MID were ½ standard deviation (SD) = 0.40, standard error of the mean (SEM) = 0.28 for the Cat QoL domain and ½SD = 0.38, SEM = 0.30 for the Owner QoL domain. For the Cat and Owner QoL domains, the correlation weighted average anchor-based responder definitions exceeded these distribution-based estimates, indicating these responder definitions represent true score change and exceed measurement error.

## 4 Discussion

The FeOA-QoL-TS-A was developed, following best practices and regulatory guidance for COA development (35, 41–43), to address the unmet need for feline OA-specific QoL measures. Results of this study support the FeOA-QoL-TS-A as fit-for-purpose for the assessment of the impact of OA on cat QoL, the impact of cat OA on owner QoL, and owner satisfaction with OA treatments. The instrument was developed to provide flexibility in the assessment of these domains, allowing it to be administered either in its entirety or as independent domain-specific scores, depending on user objectives. This intended use was supported by confirmation of the three-factor domain structure (Cat QoL, Owner QoL, Treatment Satisfaction domain scores) with acceptable CFA model fit, together with supportive item-level analyses (i.e., multi-trait analyses) and qualitative interview evidence (i.e., hypothesized conceptual model).

The FeOA-QoL-TS-A includes key concepts relevant to owners of cats with OA, as reported in the literature (2, 37) and by owners during the qualitative interviews. The most relevant Cat QoL items included cat-related impacts on mobility (e.g., difficulty jumping up/down) and energy (e.g., active). This aligns with the literature as mobility has been identified as a key domain in cats with OA (2), with impacts on mobility being some of the most frequently reported owner-perceived symptoms (3). The most relevant Owner QoL items included owner-related impacts on emotional wellbeing (e.g., sadness/depression and worry/anxiety) and ADL (e.g., increased caregiver responsibilities and reduced interaction/altered bond with cat). This aligns with the literature as greater caregiver burden is associated with depression/anxiety, as well as perceived challenges to daily routines (26). Items endorsed by fewer owners were retained where considered clinically important or potentially relevant to cats with more moderate or severe OA, including sensitivity to handling or reduced willingness to be touched or picked up, difficulty toileting, and owner-experienced sleep disruption (62). The most relevant Treatment Satisfaction items included owner-perceived satisfaction with specific treatment aspects (e.g., treatment frequency, administration or financial costs), indicating that these concepts may be associated with greater owner perceived burden regarding treatment management. The qualitative interviews confirmed that most items included were well-understood and considered relevant to at least half of the sample, providing evidence to support the content validity of the instrument. Conceptual saturation was achieved for owner QoL and narrowly missed for cat QoL, although all cat QoL concepts identified had been reported in the first set of interviews, supporting that key concepts were included in the instrument (47).

The study results provided strong reliability and validity evidence to support the psychometric properties of the resulting 20-item FeOA-QoL-TS-A and each independent domain-specific score. This supports that the instrument can accurately and reliably assess cat QoL, owner QoL, and owner treatment satisfaction with cat OA treatment, respectively, over time (56, 63–65). All domain scores demonstrated good internal consistency and test–retest reliability. When examining test–retest reliability, it was expected that Treatment Satisfaction domain scores would remain stable throughout the study, given the administered treatment remained unchanged. The results confirmed this stability, with strong test–retest reliability coefficients supporting score consistency. The Cat and Owner QoL domain scores correlated strongly with other cat and owner QoL measures assessing similar constructs (validated VetMetrica™ HRQoL instrument and newly developed FeOA global impression items), supporting convergent validity of these FeOA-QoL-TS-A domain scores. This was not assessed for the Treatment Satisfaction domain. Known groups comparisons provided strong evidence that the instrument can discriminate between groups of cat-owner pairs who differ in OA-specific overall cat QoL and owner QoL, as measured by global impression item responses. Evidence of the instrument’s ability to detect improvement over time was observed for the Cat QoL and Owner QoL domain scores, when change groups were defined using the global impression items. This supports the sensitivity of the instrument to detect improvements in cat and owner QoL over time. Additionally, anchor-based meaningful change analyses indicated within-group responder definitions for the Cat QoL (−1.0) and Owner QoL (−0.9) domain scores.

While results of this research are positive, it is important to acknowledge the limitations. For Stage 1, although there was good representation of different demographic characteristics, the sample was small (*N* = 8), with underrepresentation of male and lower-educated participants. For Stage 2, owner demographic and clinical characteristic data were not collected. This limits understanding of the representativeness of the owner samples. Consistent with Stage 1, the Stage 2 sample (cats and owners) was derived from individuals who presented their affected cats to general veterinary practice. Thus, the sample reflects real-world populations and is reflective of owners of cats with OA in veterinary practice in real-word settings. The cat samples represented in both stages of the research were senior [>10 years; mean ages: 15.2 (qualitative) and 13.6 (quantitative) years], which is expected given OA is more frequently diagnosed in cats >12 years (6, 13, 14). Additionally, cat OA severity was only captured in Stage 1, which limits interpretation of item performance across severity levels in Stage 2, for example, if floor and ceiling effects were impacted by differences in disease severity. Further research is recommended to explore any potential differences among groups who differ in OA severity.

Attrition at the final timepoint was higher than expected, possibly due to study length, cat mortality, or COVID-19-related disruptions impacting owners’ ability to attend veterinary practice for routine treatment monitoring appointments. Nonetheless, a sample size deemed sufficient for the psychometric analyses was attained. Given the known clinical effectiveness of Frunevetmab (23), expectation bias may have influenced the results, with owners preconceived expectations influencing their responses.

OA diagnosis was based on veterinary clinical judgment (detailed medical history, conversations with owner(s), and physical examination) without mandatory radiographic confirmation. As no imaging method was required, all cats had a presumed diagnosis of OA. Although this approach cannot definitively exclude other differential diagnoses that share similar symptoms (e.g., infectious arthritis), diagnosis of OA is frequently presumptive in veterinary practice (66). While imaging is useful for providing a definitive clinical diagnosis, it has limitations in detecting early OA and correlating with clinical signs (12–14). Physical examinations for OA diagnosis in cats can also present challenges (e.g., clinical signs less obvious in cats than dogs, reactions from cats regardless of symptoms), which may impact the reliability of this diagnostic method at distinguishing between OA and non-OA cats (1, 67). Due to the challenges in diagnosing OA in veterinary practice, behavioral changes observed and reported by owners are also key contributors to OA diagnosis (3, 68). Despite the noted limitations, the method of diagnosing OA in this study was deemed suitable as it reflected real-world veterinary settings where treatment decisions are typically based on veterinary judgement without extensive diagnostic tests. This aligns with the setting of the research study and the intended context of use for the FeOA-QoL-TS-A; therefore, this approach is not considered to impact the application of this research. The specific findings that supported the veterinarian’s clinical judgment decision were not collected in this research, limiting insights into how this was determined and replicability of the sample. Future research should ensure this is documented and incorporate confirmatory diagnostics (e.g., imaging) to address the highlighted diagnostic issues and improve diagnostic accuracy.

Finally, the draft FeOA-QoL-TS-A was debriefed in pen and paper format during the qualitative interviews and completed in electronic format in the phase 4 field study for psychometric evaluation. While it is recognized that generating evidence of equivalence between paper and electronic versions of the instrument is best practice, this was not deemed necessary given only minor changes were required for migration of the FeOA-QoL-TS-A to an eCOA format (69) and the response options used in the instruments were compatible with migration. In addition, the likely measurement error created by changing the mode of assessment was considered to be too small to affect the assessment of the concept of interest (41, 70, 71).

To the authors’ knowledge, the FeOA-QoL-TS-A is the first and only validated instrument for use in cats with OA that allows for the comprehensive assessment of cat QoL, owner QoL, and treatment satisfaction within a single instrument, addressing a critical measurement gap. The three-domain structure provides a standardized way of assessing these concepts in veterinary clinical practice (in-person or remotely; see Data Availability Statement for information on accessing FeOA-QoL-TS-A). Specifically:

- The Cat QoL domain assesses the impact of OA on cat QoL. It supports monitoring of OA-related impacts, informing treatment decisions, and improving disease management. Its use in clinical settings may enhance communication between veterinarians and owners, helping to overcome the under-recognition and underdiagnosis of cat OA. It also offers a standardized method for assessing treatment efficacy, supporting clinical endpoints and demonstrating treatment value.
- The Owner QoL domain assesses the impact of cat OA on owner QoL, capturing the broader impact of cat OA on owners. It can be used to facilitate discussions around treatment benefit for owner QoL, informing treatment decision making. As chronic diseases in companion animals, such as OA in cats, negatively impact owner QoL (25–28), this domain will help to provide a more holistic view of the overall impact of cat OA and improve recognition of the wider impacts on owner QoL.
- The Treatment Satisfaction domain assesses owners’ satisfaction with their cat’s OA treatment. It provides insight into owners’ perceptions of OA treatment and can be used to inform treatment selection and support adherence. It can also guide product development by identifying preferences around administration and dosing and help stakeholders communicate the value of treatment interventions.

Together, these domains offer a robust, flexible, and clinically important tool for improving the assessment and management of cat OA, enhancing outcomes for both cats and their owners. Future research could explore the implementation of the FeOA-QoL-TS-A in veterinary clinical practice, alongside its potential applications (e.g., using it to differentiate between the efficacy of different OA treatments or exploring differences between OA severities).

## 5 Conclusion

This study provides robust evidence that the final 20-item FeOA-QoL-TS-A is a reliable and valid tool for assessing cat QoL, owner QoL, and treatment satisfaction in the context of feline OA. Its domain structure allows for use of the full instrument or individual domains, depending on the assessment objective. This versatility supports its application in veterinary decision-making, stakeholder communications in pain management, and as a standardized endpoint in future clinical research. By addressing a critical measurement gap, the FeOA-QoL-TS-A offers a comprehensive and practical solution for evaluating the multifaceted impacts of OA in cats and owners.

## Data Availability

The raw data supporting the conclusions of this article will be made available by the authors, without undue reservation.

## Supplementary material

SUPPLEMENTARY FIGURE 1 FeOA-QoL-TS-A development overview.
