# Supplementary material for: Development and validation of a Quality of Life and Treatment Satisfaction measure in feline osteoarthritis
Source: Front Vet Sci. 2026 Aug 7;13:1774034. doi: 10.3389/fvets.2026.1774034 (PMC13495205; doi:10.3389/fvets.2026.1774034)
Supplement: Supplementary file 1 [file Data_Sheet_1.PDF]

	Developed based on literature review and conceptual model	Updates made following review of literature and conceptual model	Updates made based on round 1 interview findings (n=4)	Updates made based on round 2 interview findings (n=4)	Updates made based on psychometric validation
**Cat Quality of life (QoL)**	• **17 draft items** assessing cat QoL.	• 2 pairs of items assessing similar concepts were **merged** (‘grooming’, ‘pain expression’) to avoid overweighting concepts; removing 2 items.<br>• 1 item **added** (‘lying down’) based on review.<br>• 3 items assessing multiple distinct concepts (‘moving/walking’, ‘jumping up/down’, ‘climbing up/down’) were **split** into separate items; forming 3 additional items.	• 3 pairs of items assessing similar concepts **merged** into 3 single items (‘jumping up/down’, ‘climbing up/down’, ‘getting up/lying down’); removing 3 items.	• 5 items **removed** (‘visiting usual places’, ‘sleep’, ‘appetite’, showing distress, ‘has been around’) due to conceptual overlap, inability to distinguish cats with OA, and unclear conceptual relevance.<br>• 2 items (‘jumping up/down’, ‘climbing up/down’) **split** into single items due to need to assess up/down movements separately; forming 2 additional items.	• 2 items **removed** (‘jumping down’, ‘climbing up’) due to item redundancy.<br>• 1 items **removed** (‘distressed when touched or held’) due to poor psychometric performance and clinical relevance.
**Owner QoL**	• **6 draft items** assessing owner QoL.	• 3 items **removed** as the concept was unlikely to improve with treatment (e.g., guilt about cat’s pain) or due to conceptual overlap with other items.<br>• 1 item assessing multiple distinct concepts **split** into separate items (‘sad’, ‘interactions’); forming 1 additional item.<br>• 2 items **added** (‘worry’ and ‘travel to the vets’) based on review.	• 2 items **added** (‘ability to leave home’ ‘sleep’) following round 1 interim analysis.	• 1 item **removed** (‘travel to the vets’) due to lack of conceptual relevance.	• 1 item **removed** (‘clean up after cat’) based on poor psychometric performance and consideration of qualitative findings.
**Treatment Satisfaction**	• **7 draft items** assessing owner satisfaction with OA treatment.	• 4 items **removed** as they were better captured by other items or were not considered appropriate.<br>• 2 items **added** (‘treatment frequency’ and ‘overall treatment satisfaction’) based on review.<br>• 2 items assessing similar concepts (treatment administration) **merged** into a single item to avoid overweighting the concept; removing 1 item.	• 1 item **added** (‘financial impact’) following round 1 interim analysis.	• **No changes** made.	• 1 item **removed** (‘fitting treatment into daily life’) based on poor psychometric performance.
	**30** draft items initially developed	**29-item** FeOA-QoL-TS (v1_0) debriefed in round 1 interviews	**29-item** FeOA-QoL-TS (v2_0) debriefed in round 2 interviews	**25-item** FeOA-QoL-TS (v3_0) taken forward for psychometric validation	Final **20-item** FeOA-QoL-TS (v4_0) confirmed
