# Supplementary material for: Development and validation of a Quality of Life and Treatment Satisfaction measure in feline osteoarthritis
Source: Front Vet Sci. 2026 Aug 7;13:1774034. doi: 10.3389/fvets.2026.1774034 (PMC13495205; doi:10.3389/fvets.2026.1774034)
Supplement: Supplementary file 2 [file Data_Sheet_2.PDF]

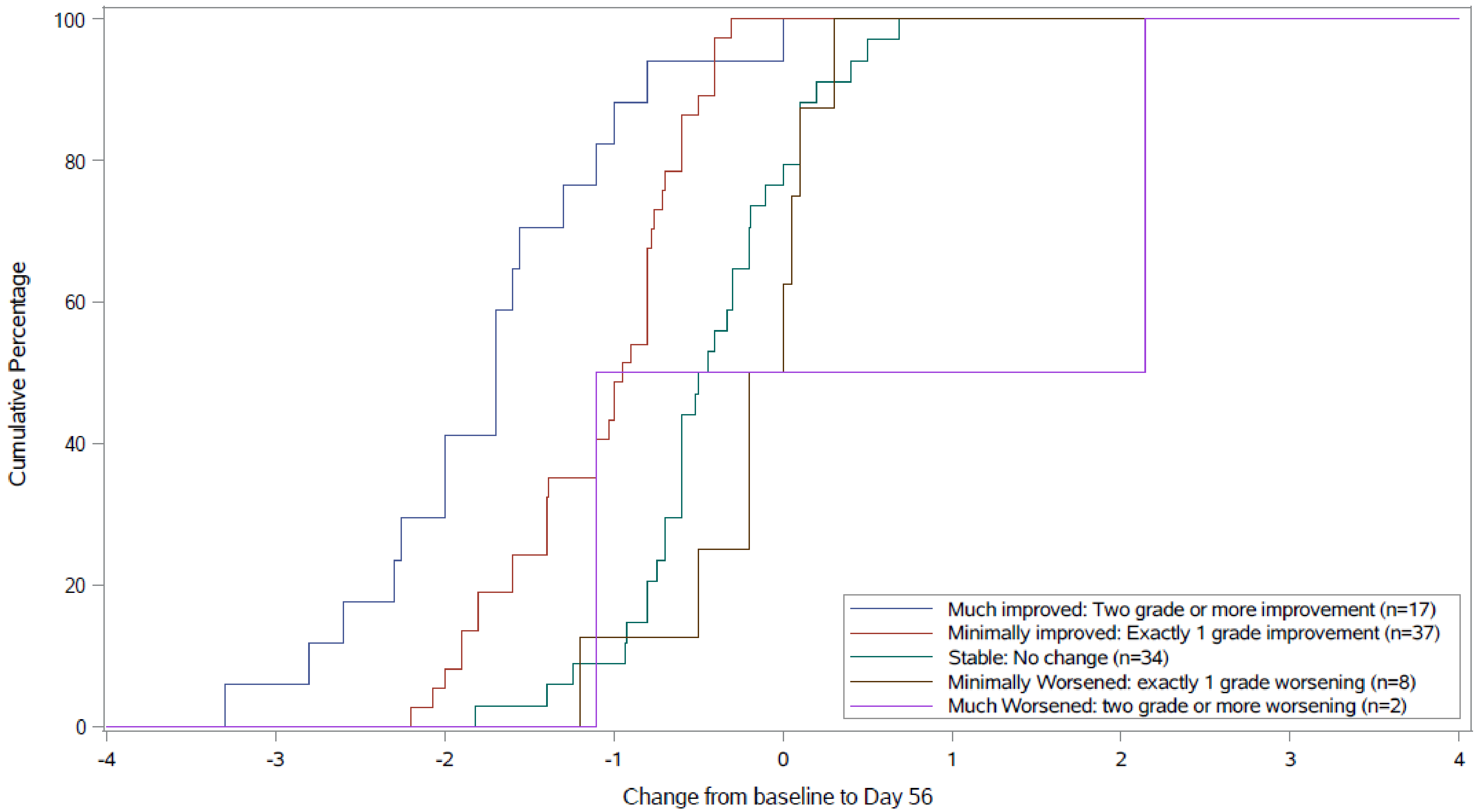

Supplementary Figure 2. Cumulative distribution function of Cat QoL domain score change by OGIC-QoL anchor group
