# Supplementary material for: Development and validation of a Quality of Life and Treatment Satisfaction measure in feline osteoarthritis
Source: Front Vet Sci. 2026 Aug 7;13:1774034. doi: 10.3389/fvets.2026.1774034 (PMC13495205; doi:10.3389/fvets.2026.1774034)
Supplement: Supplementary file 4 [file Table_1.DOCX]

| **Supplementary Table 1. Cat demographic characteristics for each sample** | | |
| --- | --- | --- |
| **Sample characteristics** | **Qualitative interviews**  **(*n*=8)** | **Validation study**  **(*n*=139)** |
| Age of cat (years)  Mean (SD) | 15.2 (2.03) | 13.6 (3.19) |
| Sex of cat, n (%)  Female  Neutered female  Male  Neutered male | 1 (12.5%)  -*  7 (87.5%)  -* | 16 (11.5%)  54 (38.8%)  10 (7.2%)  59 (42.4%) |
| Weight of cat (Kg)  Mean (SD) | -* | 4.5 (1.02) |
| Breed of cat, n (%)  Mixed breed†  Breed unknown  Purebred‡ | 5 (62.5%)  2 (25.0%)  1 (12.5%) | - ^*^  - ^*^  - ^*^ |
| Months since OA diagnosis  Mean (range) | 46.06 (3-84) | -* |
| Owner-rated current health status of cat  Fair  Very good  Good  Between Good and Fair  Poor | 3 (37.5%)  2 (25.0%)  1 (12.5%)  1 (12.5%)  1 (12.5%) | -*  -*  -*  -^*^  -* |
| Owner-rated current OA severity of cat  Mild  Moderate  Severe | 4 (50.0%)  3 (37.5%)  1 (12.5%) | -*  -*  -* |
| Previous illness other than OA  Yes  No | 6 (75.0%)  2 (25.0%) | -*  -* |
| ^*^Information not collected. †Includes domestic short hair. ‡Includes Burmese. | | |
